# Thalamo-cortical neural mechanism of sodium salicylate-induced hyperacusis and anxiety-like behaviors

**DOI:** 10.1038/s42003-024-07040-5

**Published:** 2024-10-18

**Authors:** Jingyu Chen, Xueru Wang, Zijie Li, Hui Yuan, Xuejiao Wang, Yang Yun, Xu Wu, Pingting Yang, Ling Qin

**Affiliations:** 1 Department of Physiology, School of Life Sciences, China Medical University, Shenyang, China; 2 Laboratory of Hearing Research, School of Life Sciences, China Medical University, Shenyang, China; 3 Department of Nephrology, Shengjing Hospital of China Medical University, Shenyang, China; 4 Department of Forensic Pathology, China Medical University School of Forensic Medicine, Shenyang, China; 5 Department of Rheumatology and Immunology, The First Hospital of China Medical University, Shenyang, China

**Keywords:** Cortex, Thalamus, Prefrontal cortex

## Abstract

Tinnitus has been identified as a potential contributor to anxiety. Thalamo-cortical pathway plays a crucial role in the transmission of auditory and emotional information, but its casual link to tinnitus-associated anxiety remains unclear. In this study, we explore the neural activities in the thalamus and cortex of the sodium salicylate (NaSal)-treated mice, which exhibit both hyperacusis and anxiety-like behaviors. We find an increase in gamma band oscillations (GBO) in both auditory cortex (AC) and prefrontal cortex (PFC), as well as phase-locking between cortical GBO and thalamic neural activity. These changes are attributable to a suppression of GABAergic neuron activity in thalamic reticular nucleus (TRN), and optogenetic activation of TRN reduces NaSal-induced hyperacusis and anxiety-like behaviors. The elevation of endocannabinoid (eCB)/ cannabinoid receptor 1 (CB1R) transmission in TRN contributes to the NaSal-induced abnormalities. Our results highlight the regulative role of TRN in the auditory and limbic thalamic-cortical pathways.

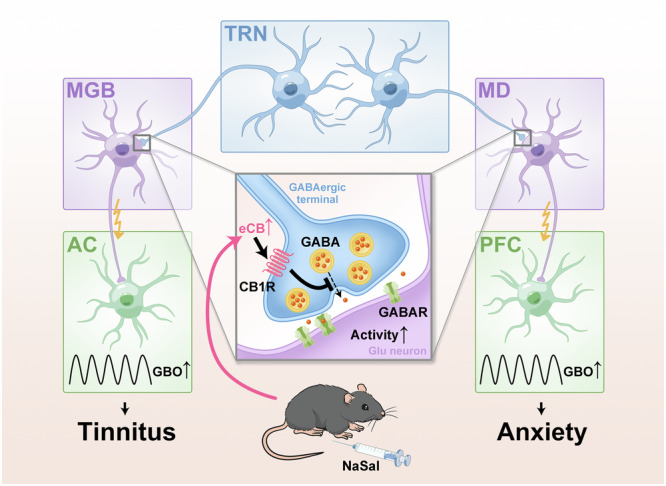

## Abstract (web summary)

Sodium salicylate enhances endocannabinoid signaling, reducing GABAergic activity in the thalamic reticular nucleus. This increases gamma oscillations in the auditory cortex and prefrontal cortex, causing hyperacusis and anxiety-like behaviors.

## Introduction

Anxiety disorders rank among the most prevalent and debilitating mental health conditions worldwide^1,2^, with a global prevalence between 3.8% and 25%^3,4^. Stressful life events are a major contributor to anxiety disorders^5^, and tinnitus, the phantom perception of sound without a corresponding external acoustic stimulus, is a common underlying factor for stress. Persistent tinnitus can cause a state of chronic stress and the stress can further aggravate tinnitus in turn^6,7^. Several clinical studies have indicated that individuals with tinnitus exhibit higher rates of anxiety disorders compared to the general population^8–11^. Additionally, the lifetime incidence of tinnitus significantly increases in individuals with anxiety disorders^12^. Although the link between tinnitus as a stressful factor and anxiety disorders was clear, the mechanism underlying this association has remained elusive.

Tinnitus is often triggered by hearing damage, but cochlear ablation or auditory nerve section never eradicates the perception of tinnitus^13^. This suggests that the generation of tinnitus involves a central mechanism. Previous studies have shown that tinnitus patients have stronger gamma band oscillation (GBO) in the auditory cortex (AC)^14^, which were associated with a reduction in the transmission of the major inhibitory neurotransmitter gamma-aminobutyric acid (GABA)^15^. In anxiety disorder patients, neural activity in the prefrontal cortex (PFC) was found to be higher during states of mental tension^16,17^. Although current data indicate the involvement of AC in tinnitus and the importance of PFC in anxiety, intensive research is still needed to address the mechanism behind the comorbidity of tinnitus and anxiety.

The thalamus traditionally functions as a ‘relay station’ for most sensory inputs on their way to cerebral cortex^18,19^, but recent studies suggest it also plays an active role in processing and integrating information^20^. Therefore, the auditory signals associated with tinnitus and the emotional signals associated with anxiety have to be processed by the thalamus before being transmitted to the cerebral cortex. The thalamus comprises two types of nuclei: first-order and higher-order thalamic nuclei^21,22^. First-order thalamic nuclei relay bottom-up sensory information to primary sensory, visual and auditory cortices^23,24^, of which the medial geniculate body (MGB) relays neural information about sound to the AC^25^. Higher-order nuclei receive their principal driver inputs from cortical layer V and transfer information among functionally related cortical regions^21,22,26^, of which the mediodorsal thalamus (MD), the largest thalamic input to the prefrontal cortex (PFC), is critical for maintaining cognitive control signals underlying emotion^27^, attention^28^, cognitive^29^, adaptive decision making^30^ and working memory^31^. The thalamic reticular nucleus (TRN), a sheet of GABAergic neurons surrounding the dorsal and lateral portions of the thalamus, provides the major inhibitory input to thalamocortical neurons (including the MGB and MD)^32–37^. As the gatekeeper of information transfer from the thalamus to the cortex, the TRN regulates information flow to maintain neural network balance and is implicated in sensory detection^38,39^, attention^40–42^, and arousal^43–45^. Nonetheless, due to the limited spatial resolution of non-invasive electroencephalogram (EEG) and functional magnetic resonance imaging (fMRI), there is insufficient evidence concerning any abnormal activity in the MGB, MD, and TRN in patients with tinnitus or anxiety disorders. Thus, despite the strong connection between the cortex and thalamus, it remains unclear whether the abnormal cortical neural activity related to tinnitus and anxiety originates from the thalamus.

To address this issue, it is necessary to conduct neurophysiological experiments on animal models of tinnitus. Sodium salicylate (NaSal)-induced tinnitus animal models have become a popular and valuable tool for pharmacological and pathological studies of the auditory system^46–48^, due to the potential of NaSal to cause adverse reactions like tinnitus and hyperacusis in humans^47,49–51^. These studies have revealed an increase in neuronal activity and GBO in both the MGB and AC, along with enhanced functional connectivity between them during NaSal-induced tinnitus^52–56^. Additionally, a reduction in presynaptic releases of GABA was reported in the AC of NaSal-treated rats^57^. Moreover, NaSal induces anxiety-like behaviors in mice, which may be associated with profound electrophysiological changes in the limbic system, such as PFC, lateral amygdala, and ventral hippocampus^58–60^. However, the specific neural mechanisms underlying the tinnitus associated anxiety in NaSal model mice remain unclear.

The primary objective of our study was to investigate the neural and molecular mechanisms underlying hyperacusis and anxiety-like behaviors of NaSal-treated mice using in vivo electrophysiological recording, fiber-optic recording, targeted recombination in active populations (TRAP), immunofluorescence, optogenetics, conditional gene knockout, and behavior tests. We found evidence supporting the idea that NaSal-induced abnormalities of auditory function and emotional state are due to the reduction in GABAergic neuronal activity in TRN, which leads to the hyperactivity of MGB-AC and MD-PFC. Moreover, an abnormal level of endocannabinoids (eCBs) acting on cannabinoid receptor 1 (CB1R) of GABAergic neurons in TRN may underly the NaSal-induced presynaptic dysfunction of TRN neurons.

## Results

### NaSal treatment induces hyperacusis and anxiety-like behaviors

**Fig. 1:**
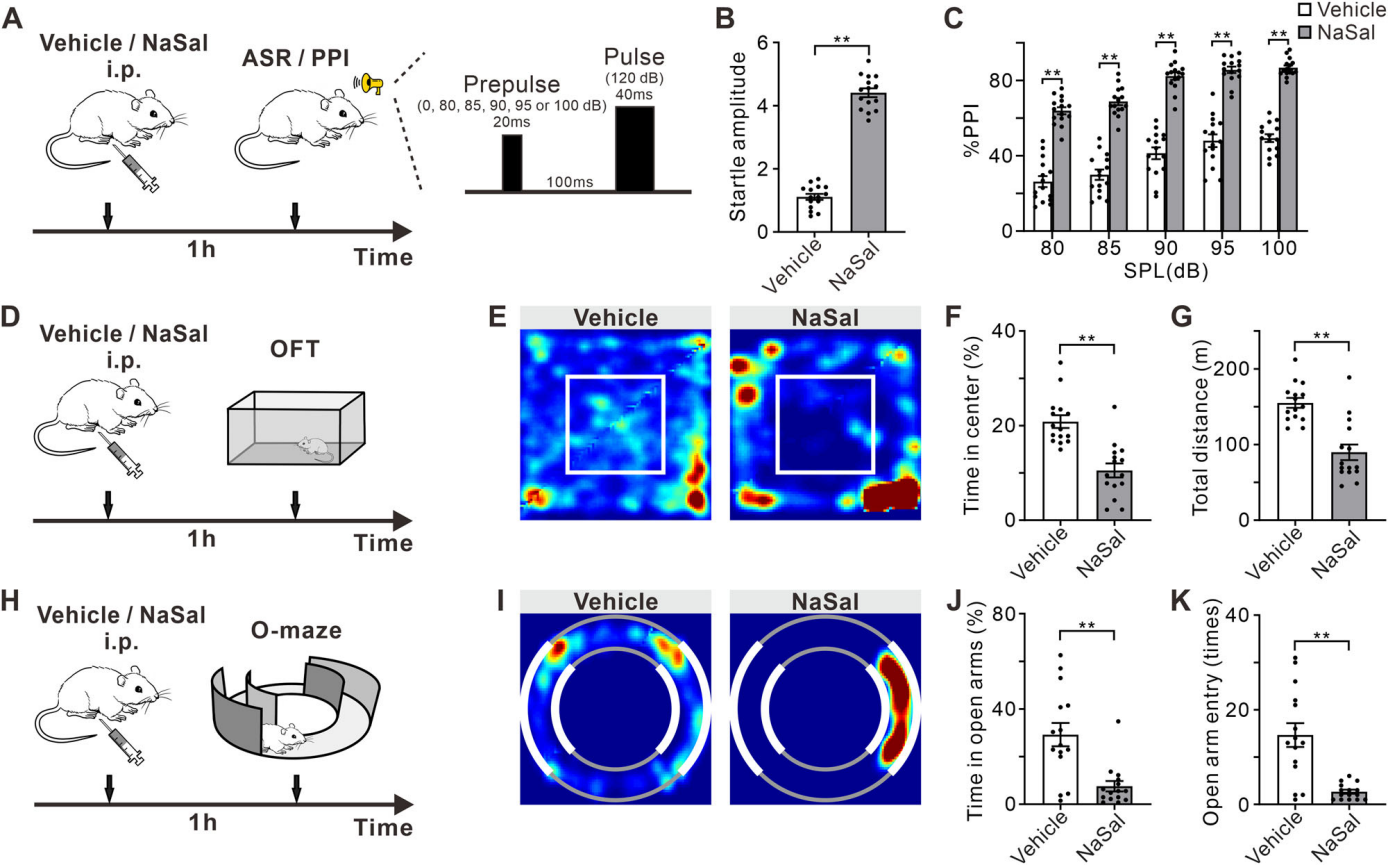
NaSal treatment induces hyperacusis and anxiety-like behaviors. **A** Timeline of drug treatment and PPI test and schematic diagram of acoustic stimuli patterns in ASR/PPI experiments. **B** Startle amplitude to acoustic stimuli at 120 dB. **C** %PPI with different volumes of prepulse from 80 to 100 dB. **D** Timeline of drug treatment and OFT. **E** Representative occupancy heatmap showing spatial location of vehicle and NaSal mice in the OFT. White rectangle marks the central area. **F** Time spent in the center of the arena. **G** Total traveled distance in the OFT. **H** Timeline of drug treatment and O-maze test**. I** Representative occupancy heatmap in the O-maze. Thick white ring parts and thin gray ring parts represent closed and open arms, respectively. **J** Time spent in open arms in the O-maze. **K** Number of entries into open arms in the O-maze.

Firstly, we performed the prepulse inhibition (PPI) test to investigate whether NaSal-treated mice exhibited hyperacusis (Fig. 1A). NaSal-treated mice significantly exhibited an increased acoustic startle response (ASR, i.e., startle amplitude) when tested with the acoustic stimuli at the sound pressure level of 120 dB (Fig. 1B, t = 19.49, df = 28, *p* < 0.0001). Analysis of %PPI of the ASR after the presentation of 80, 85, 90, 95 and 100 dB prepulse acoustic stimuli showed a main treatment effect (Fig. 1C, F _(1, 140)_ = 648.90, *p* < 0.0001), with NaSal-treated mice displaying more PPI than vehicle-treated mice, and a main effect of prepulse intensity (F _(4, 140)_ = 37.95, *p* < 0.0001). No significant treatment × prepulse intensity interaction was found (F _(4, 140)_ = 0.20, *p* = 0.9393).

Anxiety-like behavior in mice of different treatments was assessed using the open field test (OFT, Fig. 1D–G) and elevated O-maze test (O-maze, Fig. 1H–L). NaSal-treated mice spent significantly less time in the center of the arena compared to the vehicle-treated mice (Fig. 1F, t = 5.17, df = 28, *p* < 0.0001). Additionally, these mice exhibited a reduced traveled distance. (Fig. 1G, t = 5.32, df = 28, *p* < 0.0001). In the O-maze, NaSal-treated mice displayed decreased time spent in open arms (Fig. 1J, t = 4.04, df = 28, *p* = 0.0004), and fewer entries into open arms (Fig. 1K, t = 4.71, df = 28, *p* < 0.0001).

It’s worthy to note that the reduction of exploration may be due to hypo-locomotion, rather than anxious state. To validate it, we measured traveled distance of spontaneous activity in the home cage of mouse. No significant difference was found between the vehicle- and NaSal-treated groups. Because the OFT and O-maze test represented a novel environment, the reduction of traveled distance measured here reflects the reduction of novelty-induced locomotion rather than spontaneous locomotion. Therefore, our results of OFT and O-maze may reflect an anxiety-like behavior induced by NaSal treatment.

### NaSal treatment enhances Fos expression and GBO of AC and PFC

**Fig. 2:**
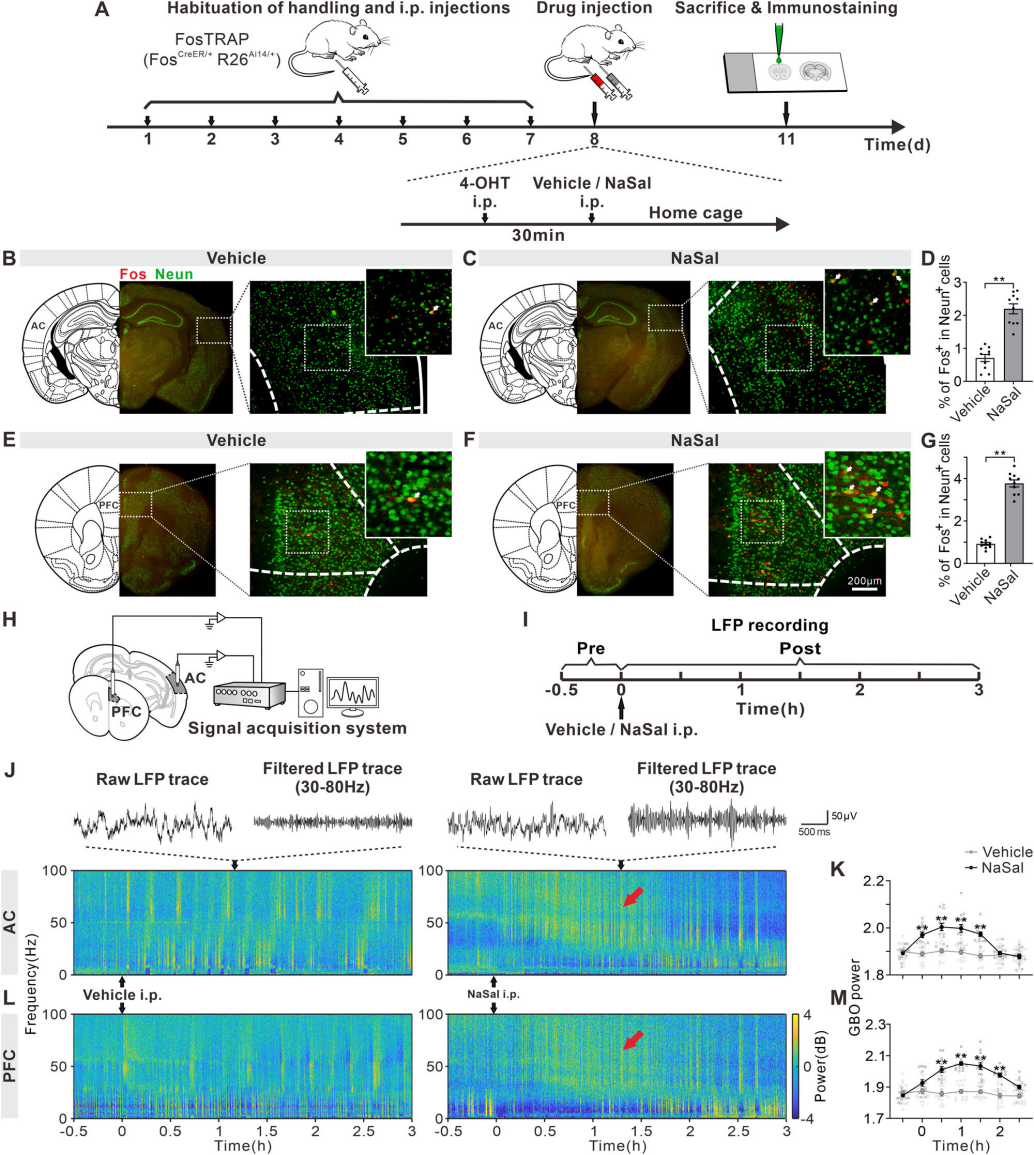
Expression of Fos and GBO in AC and PFC of NaSal-treated mice. **A** Timeline of TRAP technology for Fos and immunostaining for Neun. Fos and Neun expression observed in the AC of mice treated with vehicle (**B**) or NaSal (**C**). White arrow: NeuN^+^ FosTRAPed cells. **D** %FosTRAPed neurons in the AC. Fos and Neun expression observed in the PFC of mice treated with vehicle (**E**) or NaSal (**F**). **G** %FosTRAPed neurons in the PFC. Error bars: SEM. **p < 0.01, unpaired *t*-test, *n* = 10 mice per group. **H** Schematic diagram of electrophysiological recording. **I** Timeline of drug treatment and electrophysiological recording. Example raw traces, filtered traces and temporal-spectrograms of LFPs in the AC (**J**) and PFC (**L**). Red arrow marks the enhanced GBO. Function of mean GBO power against time in the AC (**K**) and PFC (**M**). Error bars: SEM. **p < 0.01 comparing between vehicle and NaSal groups, two-way ANOVA with Sidak multiple comparisons test, *n* = 15 mice per group.

Next, we examined the effect of NaSal treatment on AC and PFC neurons using TRAP technology for the immediate early gene product Fos (Fig. 2A). NaSal treatment significantly increased the percentage of FosTRAPed neurons (i.e., NeuN^+^ FosTRAPed cells/ total mature NeuN^+^ cells) in the AC (Fig. 2B–D, t = 8.04, df = 18, *p* < 0.0001) and PFC (Fig. 2E–G, t = 16.15, df = 18, *p* < 0.0001). We selected these Fos-positive regions as targets for subsequent experiments investigating the AC and PFC.

We further recorded the local field potentials (LFPs) in the AC and PFC from 0.5 h pre- to 3 h post-vehicle/NaSal injection (Fig. 2H, I). The temporal-spectrograms of LFP exhibit an increase of GBO power after NaSal injection (Fig. 2J, L). The mean GBO power was significantly increased in the AC from 0 to 1.5 h after NaSal injection (Fig. 2K, F _treatment (1, 28)_ = 21.10, *p* < 0.0001, F _time (6, 168)_ = 38.44, *p* < 0.0001, F _treatment × time interaction (6, 168)_ = 30.34, *p* < 0.0001), and in the PFC from 0.5 to 2 h (Fig. 2M, F _treatment (1, 28)_ = 36.53, *p* < 0.0001, F _time (6, 168)_ = 34.45, *p* < 0.0001, F _treatment × time interaction (6, 168)_ = 27.71, *p* < 0.0001). Thus, NaSal can induce neural activation and GBO increase in the AC and PFC.

### Effects of NaSal treatment on the activities of thalamus and their phase-locking with cortical GBO bursts

**Fig. 3:**
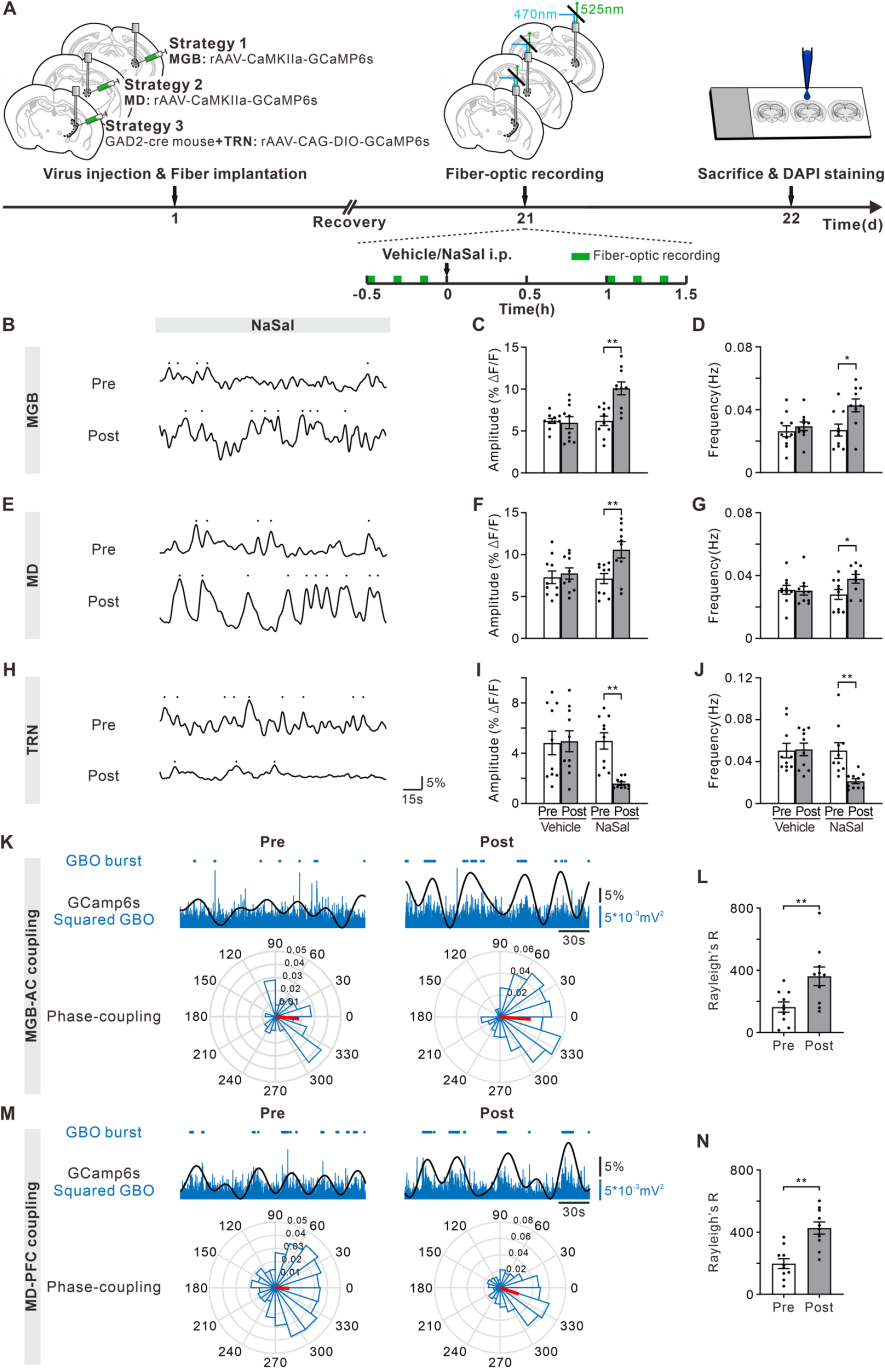
Calcium transients of glutamatergic and GABAergic neurons in the thalamus and phase-locking of MGB-AC and MD-PFC in NaSal-treated mice. **A** Timeline of the virus injection, drug treatment and fiber-optic recording. Representative calcium transient traces of the MGB (**B**), MD (**E**) and TRN (**H**) before and after NaSal injection. Dot marks a transient event higher than mean + SD of ΔF/F. **C**, **F**, **I** Effect of the vehicle (left) and NaSal (right) treatment on the amplitude of calcium transients. **D**, **G**, **J** Effect of the vehicle (left) and NaSal (right) treatment on the frequency of calcium transients. **K** Examples of calcium transients (black line) of MGB neurons and GBO (blue line) of AC. Blue dot: identified GBO burst. Polar-histogram: the phase-locking between GBO bursts of AC and calcium transients of MGB neurons. Red bar: mean vector length of phase-locking value. **L** Bar chart showing R value in Rayleigh’s test of MGB-AC phase-locking before and after NaSal injection. **M** Examples of calcium transients of MD neurons and GBO of PFC and the phase-locking between them. **N** Bar chart showing R value in Rayleigh’s test of MD-PFC phase-locking. *p < 0.05, **p < 0.01, unpaired *t*-test, n = 10 mice per group.

To further investigate the effect of NaSal on neural activities, we used fiber photometry to record the immediate calcium signals from MGB, MD, and TRN neurons (Fig. 3A). We injected rAAV-CaMKIIa-GCaMP6s into either the unilateral MGB or MD of WT mice. While CaMKIIa may also be expressed in inhibitory neurons, previous histological studies have demonstrated that the sensory thalamus of rodents is primarily composed of glutamatergic neurons, with a very small proportion of inhibitory neurons^32,61,62^. Therefore, the use of the CaMKIIa promoter to transfect the glutamatergic neurons of MGB and MD is appropriate. On the other hand, the TRN area is the sole region in the rodent thalamus where GABAergic inhibitory neurons aggregate^63^. We used rAAV-CAG-DIO-GCaMP6s injected into TRN of GAD2-cre mice to selectively transfect GABAergic neurons with GCaMP6s. After 21 days of recovery, the fiber-optic recordings were conducted at pre- and 1 h post-vehicle/NaSal injection (when NaSal-induced GBO was the most obvious). In the glutamatergic neurons of MGB, we found a significant increase in the amplitude and frequency of calcium transients between pre- and post-NaSal injection groups (Fig. 3C, t = 4.10, df = 18, *p* = 0.0007; Fig. 3D, t = 2.83, df = 18, *p* = 0.0112), but not between pre- and post-vehicle injection groups (*p* = 0.7853 and *p* = 0.4845). Similarly, the calcium transient amplitude and frequency of glutamatergic neurons in the MD was also increased after the NaSal injection (Fig. 3F, t = 2.98, df = 18, *p* = 0.0080; Fig. 3G, t = 2.38, df = 18, *p* = 0.0289). However, NaSal injection decreased the calcium transient amplitude and frequency of the TRN GABAergic neurons significantly (Fig. 3I, t = 5.10, df = 18, *p* < 0.0001; Fig. 3J, t = 3.74, df = 18, *p* = 0.0015).

Comparing with GBO bursts in the simultaneously recorded LFPs, we observed that NaSal injection enhanced the phase-locking between the GBO bursts of AC and the calcium transients of MGB neurons (Fig. 3K and L, t = 2.90, df = 18, *p* = 0.0096). The phase-locking between the GBO bursts of PFC and the calcium transients of MD neurons also increased significantly after NaSal injection (Fig. 3M and N, t = 4.53, df = 18, *p* = 0.0003). Therefore, the neural activities were decreased in the TRN by NaSal, but increased in the MGB and MD, contributing to the enhancement of GBO in the AC and PFC.

### Calcium activities of MD neurons during O-maze test

**Fig. 4:**
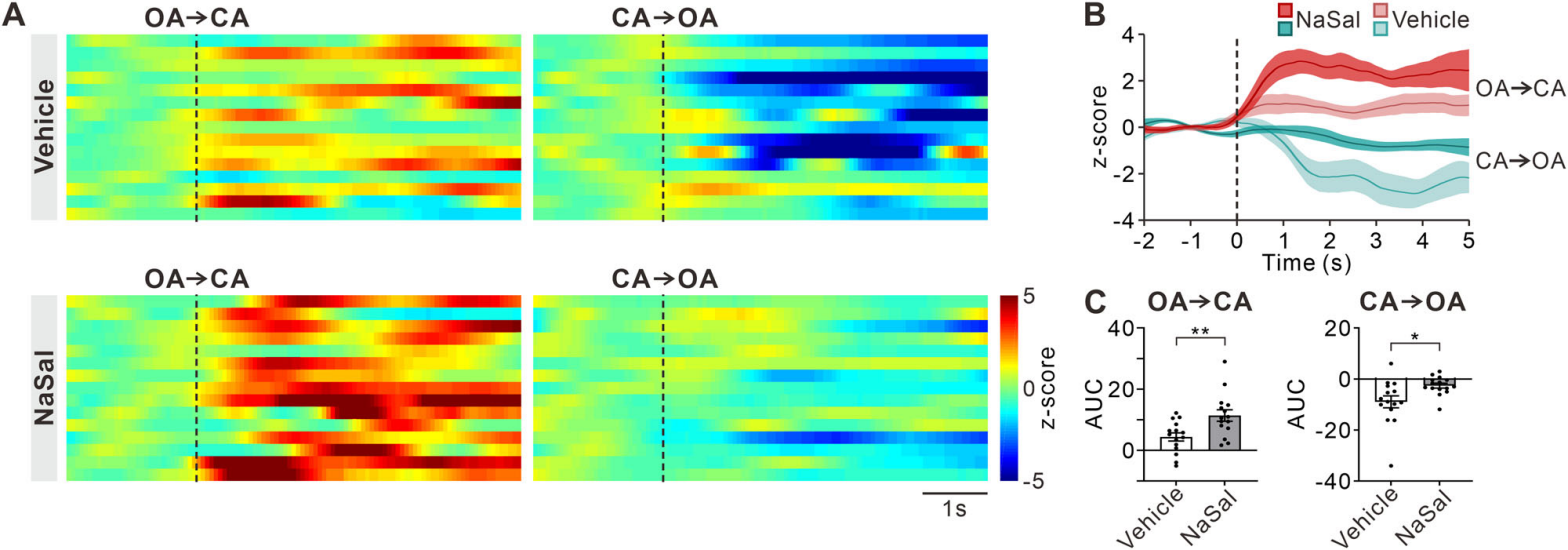
Calcium transients of MD glutamatergic neurons across arm compartments of O-Maze in NaSal-treated mice. **A** Heatmap of calcium transients between arm compartments in the O-maze apparatus. OA: open arms; CA: closed arms. **B** Line graph showing average changes in normalized calcium transients between arm compartments. Data are presented as mean ± SE. **C** Area under the curve (AUC) of normalized calcium transients. Error bars: SEM. *p < 0.05, **p < 0.01, unpaired *t*-test.

Next, to examine the association between MD neuron activity and anxiety, we conducted fiber photometry in the O-maze apparatus. We analyzed calcium signals from MD glutamatergic neurons as the mice ran across different arm compartments of the O-maze. Calcium signals of vehicle-treated mice increased when transitioning from open arms to closed arms (OA → CA), while decreased, from closed arms to open arms (CA → OA, Fig. 4A, B). Compared to vehicle-treated mice, NaSal-treated mice exhibited a more pronounced increase in calcium signals at OA → CA, but a less pronounced decrease at CA → OA. Quantitative analysis showed that the area under the curve (AUC) of calcium signals at OA → CA was significantly higher in NaSal-treated mice compared to vehicle-treated mice (Fig. 4C, t = 3.05, df = 28, *p* = 0.0050). Conversely, at CA → OA, NaSal-treated mice showed a significantly smaller reduction in AUC compared to vehicle-treated mice (t = 2.52, df = 28, *p* = 0.0176).

### Optogenetic activation of MGB-AC or MD-PFC induces changes in cortical GBO and alterations in auditory function or emotional state

**Fig. 5:**
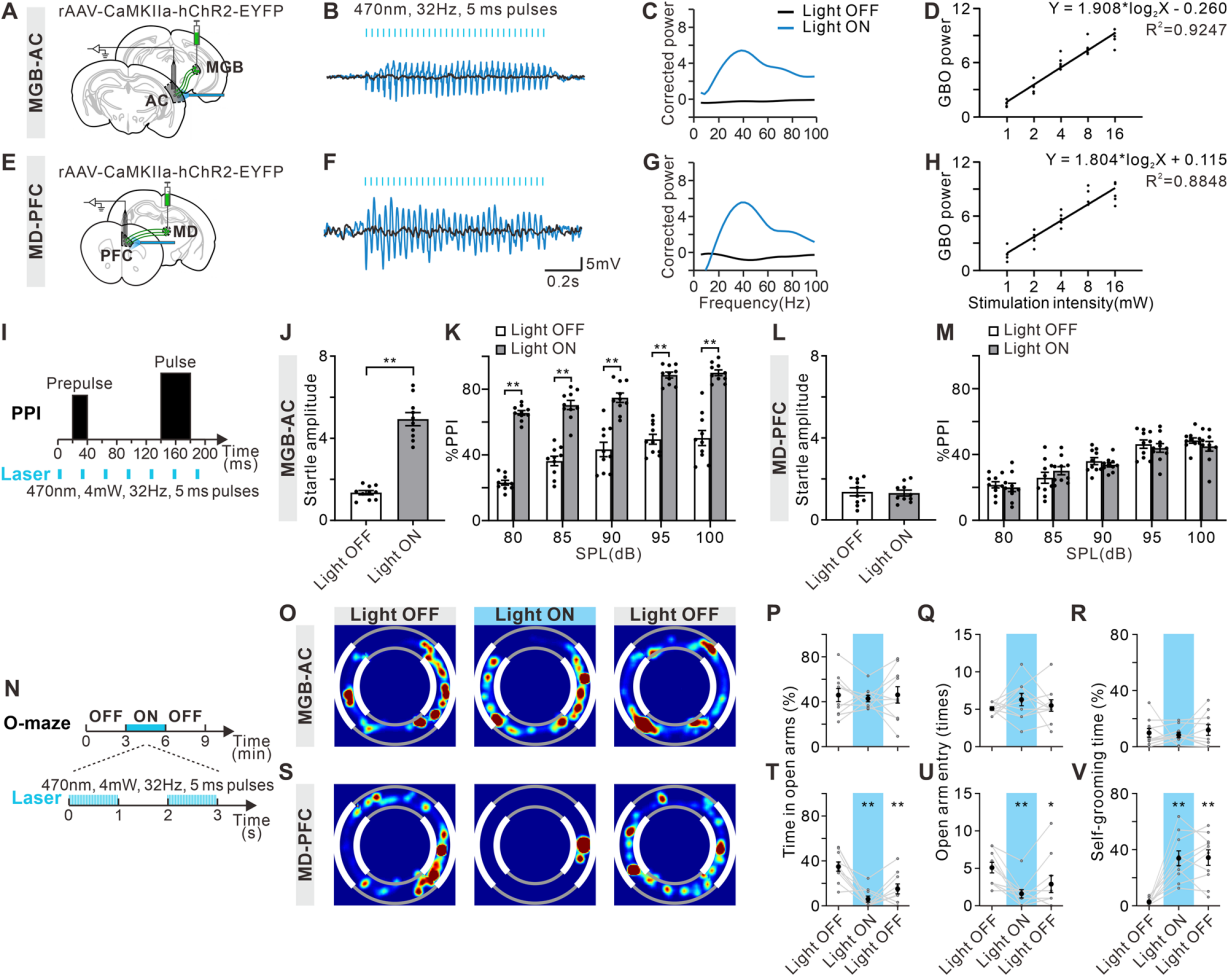
Optogenetic activation of MGB-AC or MD-PFC induces changes in cortical GBO and alterations in auditory function or emotional state. Schematic diagram of LFP recording and optogenetic activation of MGB-AC (**A**) and MD-PFC (**E**). Examples of LFP in AC (**B**) and PFC (**F**) with (blue) or without optogenetic activation (black). Power spectrum of LFP in AC (**C**) and PFC (**G**) showing an increase of GBO power (30-80 Hz) induced by optogenetic activation. **D** Relationship between GBO power of AC and optogenetic stimulation intensity of MGB-AC. Solid line shows the estimated regression curve. **H** Relationship between GBO power of PFC and optogenetic stimulation intensity of MD-PFC. **I** Schematic diagram of ASR/PPI test and optogenetic activation. Startle amplitude to acoustic stimuli at 120 dB with or without the activation of MGB-AC (**J**) and MD-PFC (**L**). **K**, **M** %PPI with different volumes of prepulse from 80 to 100 dB. Error bars: SEM. **p < 0.01, unpaired *t*-test or two-way ANOVA with Sidak multiple comparisons test, *n* = 10 mice per group. **N** Schematic diagram of O-maze test and optogenetic activation, consisting of three 3-min epochs with alternating light manipulation (OFF-ON-OFF). Representative occupancy heatmap showing spatial location in the O-maze during optogenetic activation of MGB-AC (**O**) and MD-PFC (**S**). Time spent in open arms in the O-maze test during optogenetic activation of MGB-AC (**P**) and MD-PFC (**T**). **Q**, **U** Number of entries into open arms in the O-maze test. **R**, **V** Self-grooming time in the O-maze test. *p < 0.05, **p < 0.01 comparing to the first Light OFF group, one-way ANOVA with Geisser-Greenhouse correction and Dunnett multiple comparisons test, *n* = 10 mice per group.

To further investigate the association between thalamic neural activity and cortical GBO, we recorded the LFPs of the AC or PFC in response to the optogenetic activation of MGB-AC (Fig. 5A-D) or MD-PFC (Fig. 5E–H). As illustrated by Fig. 5B, F, the rhythmic activation (470 nm, 32 Hz, 5 ms pulse width) of MGB-AC and MD-PFC effectively induced resonant LFP activity in the AC and PFC, respectively. The power spectrum of the LFP exhibited a peak around 32 Hz (Fig. 5C, G), and the GBO power increased with stimulation intensity (Fig. 5D, H).

We further examined whether optogenetic activation of the MGB-AC and MD-PFC pathways could acutely induce abnormalities in auditory and emotional function using the experimental procedures outlined in Fig. 5I, N. When the MGB-AC was optogenetically activated, mice exhibited an increase in ASR intensity to acoustic stimuli at 120 dB (Fig. 5J, t = 10.75, df = 18, *p* < 0.0001). %PPI showed a main treatment effect (Fig. 5C, F _(1, 90)_ = 416.30, *p* < 0.0001), as well as a main effect of prepulse intensity (F _(4, 90)_ = 28.21, *p* < 0.0001), but no significant treatment × prepulse intensity interaction (F _(4, 90)_ = 1.20, *p* = 0.3169). Conversely, activation of the MD-PFC did not induce any changes in ASR intensity or %PPI (Fig. 5L, t = 0.24, df = 18, *p* = 0.8098; Fig. 5M, F _treatment (1, 90)_ = 0.50, *p* = 0.4819, F _prepulse intensity (4, 90)_ = 41.82, *p* < 0.0001, F _treatment × prepulse intensity interaction (4, 90)_ = 0.81, *p* = 0.5249). MGB-AC activation did not significantly affect time spent in open arms, open arm entry times, or self-grooming time in the O-maze (Fig. 5O, P, F _(1.76, 15.84)_ = 0.15, *p* = 0.8400; Fig. 5Q, F _(1.97, 17.76)_ = 0.89, *p* = 0.4286; Fig. 5R, F _(2.00, 17.97)_ = 0.43, *p* = 0.6561). However, statistical analysis revealed a significant decrease in time spent in open arms and open arm entry times, and an increase in self-grooming time, a parameter relevant for high level of anxiety^64,65^, upon activation of the MD-PFC pathway (Fig. 5S and T, F _(1.85, 16.67)_ = 20.41, *p* < 0.0001; Fig. 5U, F _(1.97, 17.72)_ = 10.55, *p* = 0.0010; Fig. 5V, F _(1.78, 16.03)_ = 25.91, *p* < 0.0001). These anxiety-like behaviors persisted during the Light OFF period. Thus, MGB-AC and MD-PFC activation were closely related to hyperacusis and anxiety-like behaviors, respectively.

### Optogenetic activation of TRN neurons ameliorated NaSal-induced cortical GBO, hyperacusis, and anxiety-like behaviors

**Fig. 6:**
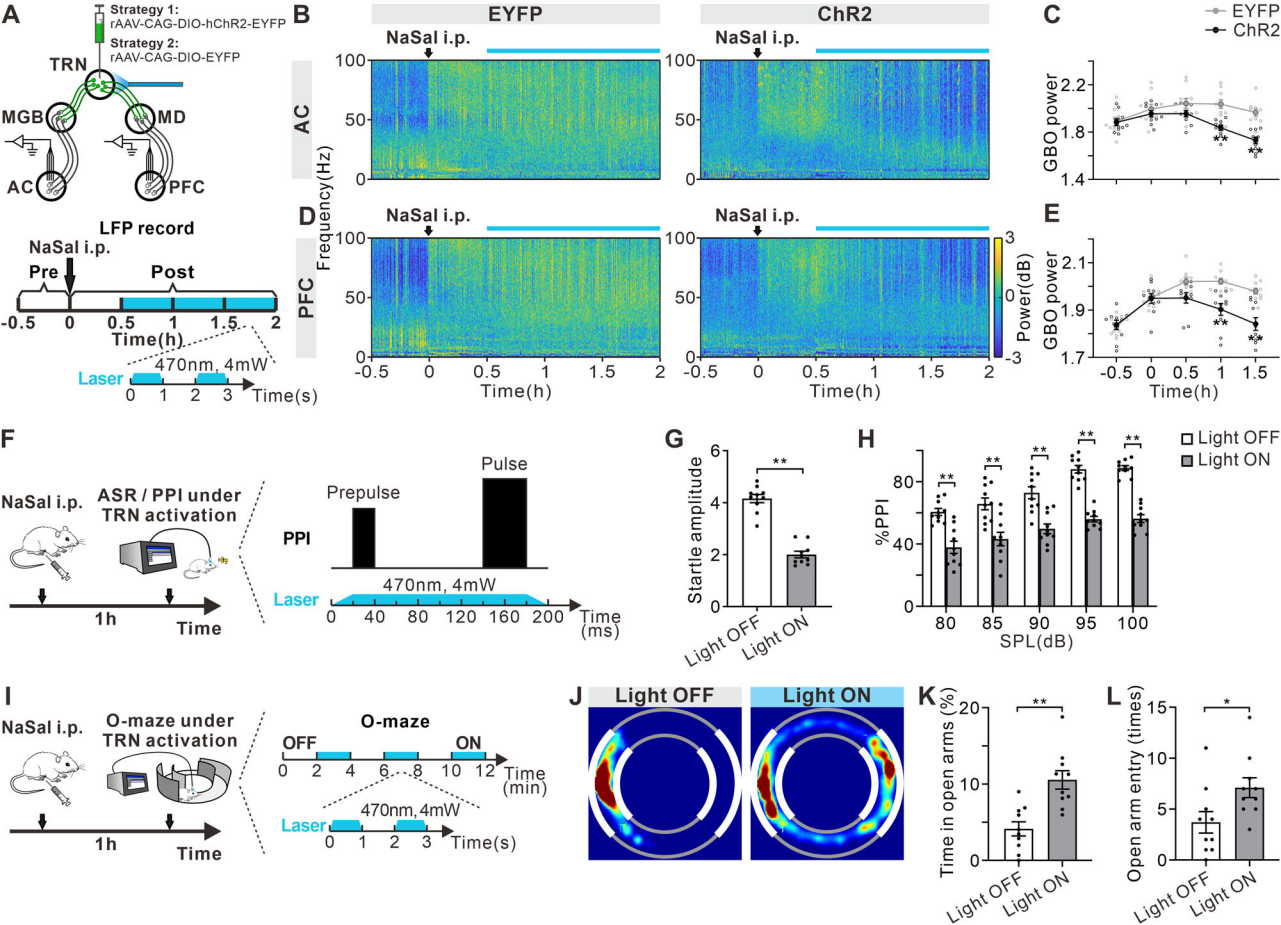
Optogenetic activation of TRN induces changes in cortical GBO and alterations in auditory function and emotional state. **A** Schematic diagram of LFP recording and optogenetic activation of TRN GABAergic neurons and timeline of drug treatment, LFP recording and optogenetic activation. Example temporal-spectrograms of LFPs in the AC (**B**) and PFC (**D**). Function of mean GBO power against time in the AC (**C**) and PFC (**E**) of NaSal-treated mice. Error bars: SEM. **p < 0.01 comparing between EYFP and ChR2 groups, two-way ANOVA with Sidak multiple comparisons test, *n* = 10 mice per group. **F** Schematic diagram of ASR/PPI test and TRN optogenetic activation. **G** Startle amplitude to acoustic stimuli at 120 dB with or without TRN activation. **H**. %PPI with different volumes of prepulse from 80 to 100 dB. **p < 0.01, unpaired *t*-test or two-way ANOVA with Sidak multiple comparisons test, *n* = 10 mice per group. **I**Schematic diagram of O-maze test and TRN optogenetic activation, consisting of six 2-min epochs with alternating light manipulation (OFF-ON-OFF-ON-OFF-ON). **J** Representative occupancy heatmap showing spatial location in the O-maze with or without TRN activation. **K** Time spent in open arms in the O-maze test. **L** Number of entries into open arms in the O-maze test. *p < 0.05, **p < 0.01, unpaired *t*-test, *n* = 10 mice per group.

Next, we conducted LFP recordings on the AC and PFC of NaSal-treated mice expressing ChR2 or EYFP in the TRN GABAergic neurons to investigate the effect of optogenetic activation of TRN neurons (Fig. 6A). The results confirmed that TRN neuron activation significantly suppressed GBO in both the AC and PFC at 1-1.5 h post NaSal injection compared to the EYFP group (Fig. 6B, C, F _treatment (1, 18)_ = 7.00, *p* = 0.0164, F _time (4, 72)_ = 83.79, *p* < 0.0001, F _treatment × time interaction (4, 72)_ = 54.19, *p* < 0.0001; Fig. 6D, E, F _treatment (1, 18)_ = 7.12, *p* = 0.0156, F _time (4, 72)_ = 102.90, *p* < 0.0001, F _treatment × time interaction (4, 72)_ = 31.16, *p* < 0.0001).

To explore the effect of TRN neural activity on auditory function, we injected mice with NaSal and subjected them to optogenetic activation of the TRN neurons and ASR/PPI tests at 1 h after the injection (Fig. 6F). Compared to the Light OFF group, NaSal-treated mice in the Light ON group exhibited significant decreases in ASR intensity and %PPI (Fig. 6G, t = 10.56, df = 18, *p* < 0.0001; Fig. 6H, F _treatment (1, 90)_ = 184.10, *p* < 0.0001; F _prepulse intensity (4, 90)_ = 22.42, *p* < 0.0001; F _treatment × prepulse intensity interaction (4, 90)_ = 1.41, *p* = 0.2383). O-maze was also performed on the NaSal-treated mice with or without activation of the TRN neurons (Fig. 6I). We found that the mice spent more time in the open arms and entered the open arms more frequently when their TRN neurons were activated (Fig. 6J and K, t = 4.23, df = 18, *p* = 0.0005; Fig. 6L, t = 2.37, df = 18, *p* = 0.0291).

### NaSal treatment increases the level of eCBs in MGB and MD

**Fig. 7:**
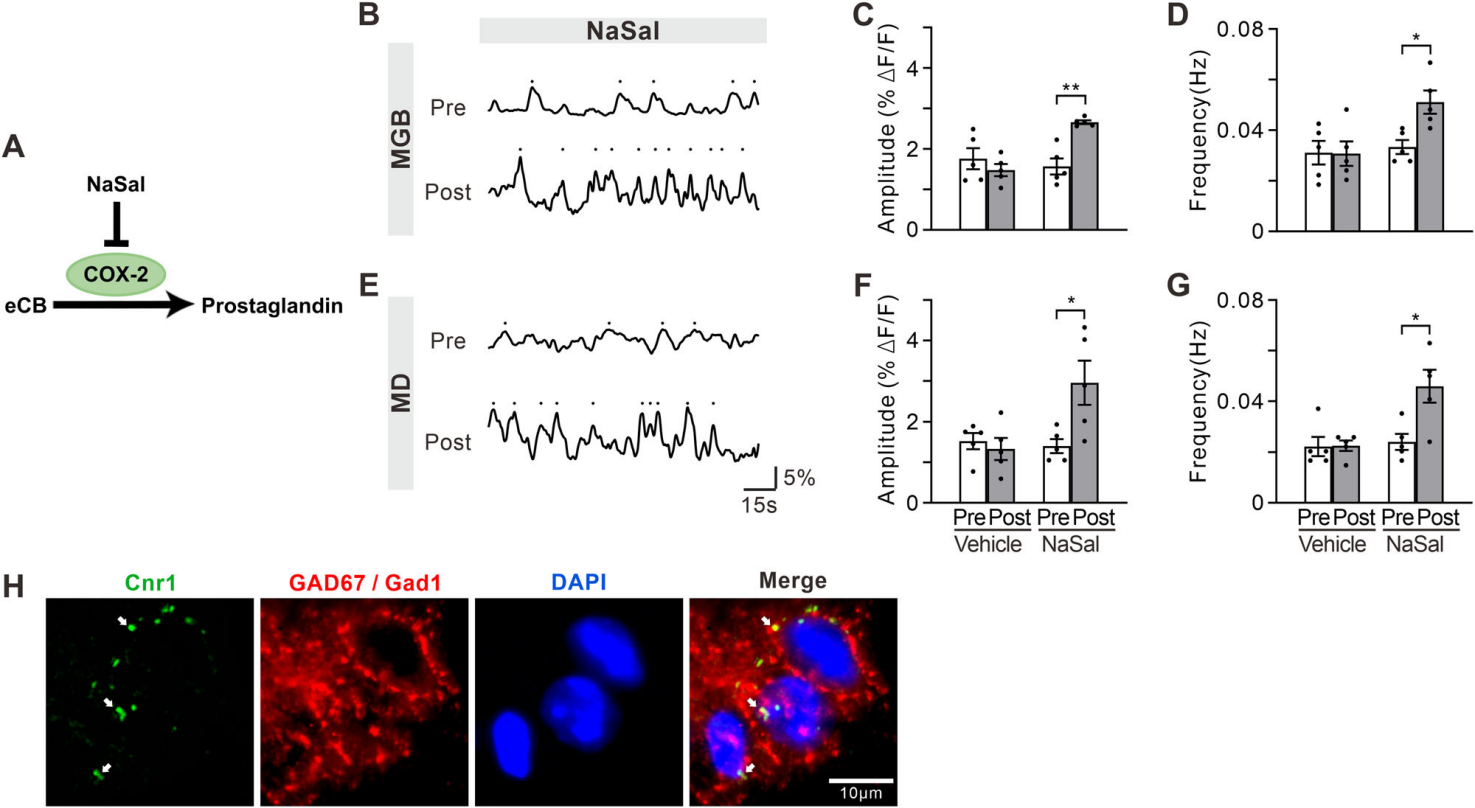
eCB levels in NaSal-treated mice. **A** Schematic diagram of an inhibiting effect of NaSal on eCB metabolism. Representative eCB2.0 fluorescence signals in the MGB (**B**) and MD (**E**) before and after NaSal injection. **C**, **F** Effect of vehicle (left) and NaSal (right) treatment on the amplitude and of eCB2.0 transients. **D**, **G** Effect of vehicle (left) and NaSal (right) treatment on the frequency of eCB2.0 transients. Error bars: SEM. **p < 0.01, unpaired *t*-test, *n* = 5 mice per group. **H** FISH of *Cnr1* mRNA and immunofluorescence of GAD67/Gad1 images. White arrow marks a Cnr1^+^ GAD67/Gad1^+^ cell.

Previous studies have shown that NaSal inhibits the activity of cyclooxygenase-2 (COX-2)^66^, which has the ability to metabolize eCBs^67^ (Fig. 7A). Therefore, NaSal treatment may elevate eCB level. We examined eCB dynamics in the MGB and MD using fiber-optic recording of eCB2.0^68^ to test this hypothesis (Fig. 7B-G). Figure 7B illustrates the representative eCB2.0 fluorescence dynamics in the MGB before and after NaSal injection. No significant change was found in the amplitude and frequency of eCB2.0 transients between pre- and 1 h post-vehicle injection (Fig. 7C, t = 0.93, df = 8, *p* = 0.3774; Fig. 7D, t = 0.06, df = 8, *p* = 0.9574). However, we observed a significant increase in the amplitude and frequency of the eCB2.0 transients after NaSal injection (Fig. 7C, t = 5.36, df = 8, *p* = 0.0007; Fig. 7D, t = 3.32, df = 8, *p* = 0.0105). Similarly, the amplitude and frequency of the eCB2.0 transients in the MD were not changed by vehicle injection (Fig. 7E and F, t = 0.57, df = 8, *p* = 0.5857; Fig. 7G, t = 0.09, df = 8, *p* = 0.9338), but showed a significant increase after NaSal injection (Fig. 7F, t = 2.72, df = 8, *p* = 0.0263; Fig. 7G, t = 3.02, df = 8, *p* = 0.0165).

In the central nervous system (CNS), eCBs generally bind to presynaptic CB1Rs to suppress neurotransmitter release^69^. We examined the mouse database in the Allen Brain Atlas and confirmed high mRNA expression of *Cnr1* (the gene encoding CB1R) in the TRN (http://mouse.brain-map.org). By using fluorescence in situ hybridization (FISH) combined with immunofluorescence in the TRN section, we found that *Cnr1* mRNA was localized in cells expressing GAD67 protein (coded by *Gad1* gene, Fig. 7H). Therefore, NaSal treatment may increase the level of eCBs in the MGB and MD, which can act on CB1R on TRN GABAergic neurons, mediating the presynaptic inhibition of GABA.

### CB1R antagonist AM251 reduced GBO and anxiety-like behaviors induced by NaSal

**Fig. 8:**
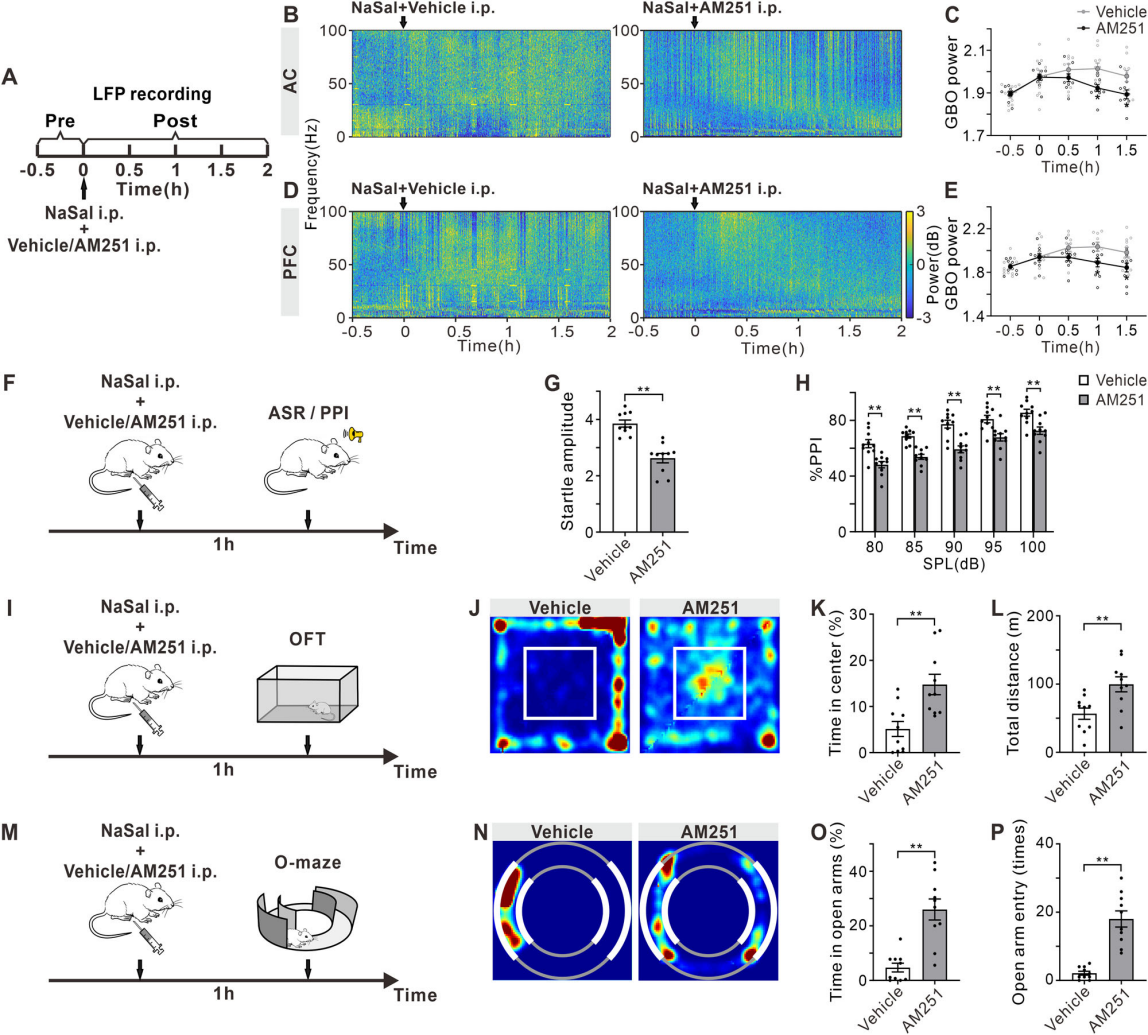
Effect of CB1R antagonist AM251 on NaSal-induced GBO and anxiety-like behaviors. **A** Timeline of drug treatment and LFP recording. Example temporal-spectrograms of LFPs in the AC (**B**) and PFC (**D**). Function of mean GBO power in the AC (**C**) and PFC (**E**) against time of mice received NaSal + vehicle/AM251 injection. Error bars: SEM. *p < 0.05 comparing between vehicle and AM251 groups, two-way ANOVA with Sidak multiple comparisons test, *n* = 10 mice per group. **F** Timeline of drug treatment and ASR/PPI test. **G** Startle amplitude to acoustic stimuli at 120 dB. **H** %PPI with different volumes of prepulse from 80 to 100 dB. **I** Timeline of drug treatment and OFT. **J** Representative occupancy heatmap showing spatial location in the OFT. **K** Time spent in the center of the arena. **L** Total traveled distance in the OFT. **M** Timeline of drug treatment and O-maze test. **N** Representative occupancy heatmap showing spatial location in the O-maze. **O** Time spent in open arms in the O-maze. **P** Number of entries into open arms in the O-maze. Error bars: SEM. **p < 0.01, unpaired *t*-test or two-way ANOVA with Sidak multiple comparisons test, *n* = 10 mice per group.

To investigate whether NaSal induced GBO increase and anxiety-like behaviors through a CB1R-dependent mechanism, we repeated the LFP recordings and behavioral tests combining AM251 (CB1R antagonist) or vehicle (saline) with NaSal injection. In the LFP recording experiments (Fig. 8A–E), the mean GBO power in the AC decreased significantly from 0.5 to 1.5 h after NaSal injection in the mice with AM251 treatment (Fig. 8C, F _treatment (1, 18)_ = 2.44, *p* = 0.1356, F _time (4, 72)_ = 26.28, *p* < 0.0001, F _treatment × time interaction (4, 72)_ = 9.05, *p* < 0.0001). Similar results were also observed in the PFC (Fig. 8E, F _treatment (1, 18)_ = 2.80, *p* = 0.1118, F _time (4, 72)_ = 12.49, *p* < 0.0001, F _treatment × time interaction (4, 72)_ = 6.18, *p* = 0.0002).

In the ASR/PPI tests (Fig. 8F-H), AM251 treatment significantly decreased the ASR intensity in the NaSal mice, compared with vehicle treatment (Fig. 8G, t = 5.87, df = 18, *p* < 0.0001). %PPI was also decreased by the AM251 treatment (Fig. 8H, F _treatment (1, 90)_ = 91.28, *p* < 0.0001, F _prepulse intensity (4, 90)_ = 29.86, *p* < 0.0001, F _treatment × prepulse interaction (4, 90)_ = 0.35, *p* = 0.8404). In the OFT (Fig. 8I-L), the mice received AM251 treatment spent significantly more time in the center of the arena (Fig. 8J and K, t = 3.49, df = 18, *p* = 0.0026) and traveled a longer distance compared to the vehicle-treated mice (Fig. 8L, t = 3.13, df = 18, *p* = 0.0058). One point should be clarified that mice in the vehicle group, which received both NaSal and vehicle treatments, exhibited more pronounced anxiety-like behaviors (reduced center time and total distance traveled) compared to those receiving a single NaSal treatment as shown in Fig. 1. This was likely due to being handled and injected twice within a short period. Both the AM251 and vehicle groups underwent similar handling and injection procedures twice during this experiment, thus this procedure would not produce any systemic interference on our observations of the effects of AM251. In the O-maze (Fig. 8M-P), the AM251-treated mice spent more time in the open arms (Fig. 8N and O, t = 5.10, df = 18, *p* < 0.0001) and entered more into the open arms (Fig. 8P, t = 6.52, df = 18, *p* < 0.0001). Therefore, CB1R has mediated the GBO increase of the AC and PFC and the anxiety-like behaviors induced by NaSal.

### Selective knockout of CB1R in TRN neurons alleviated NaSal-induced enhancement of GBOs and anxiety-like behaviors

**Fig. 9:**
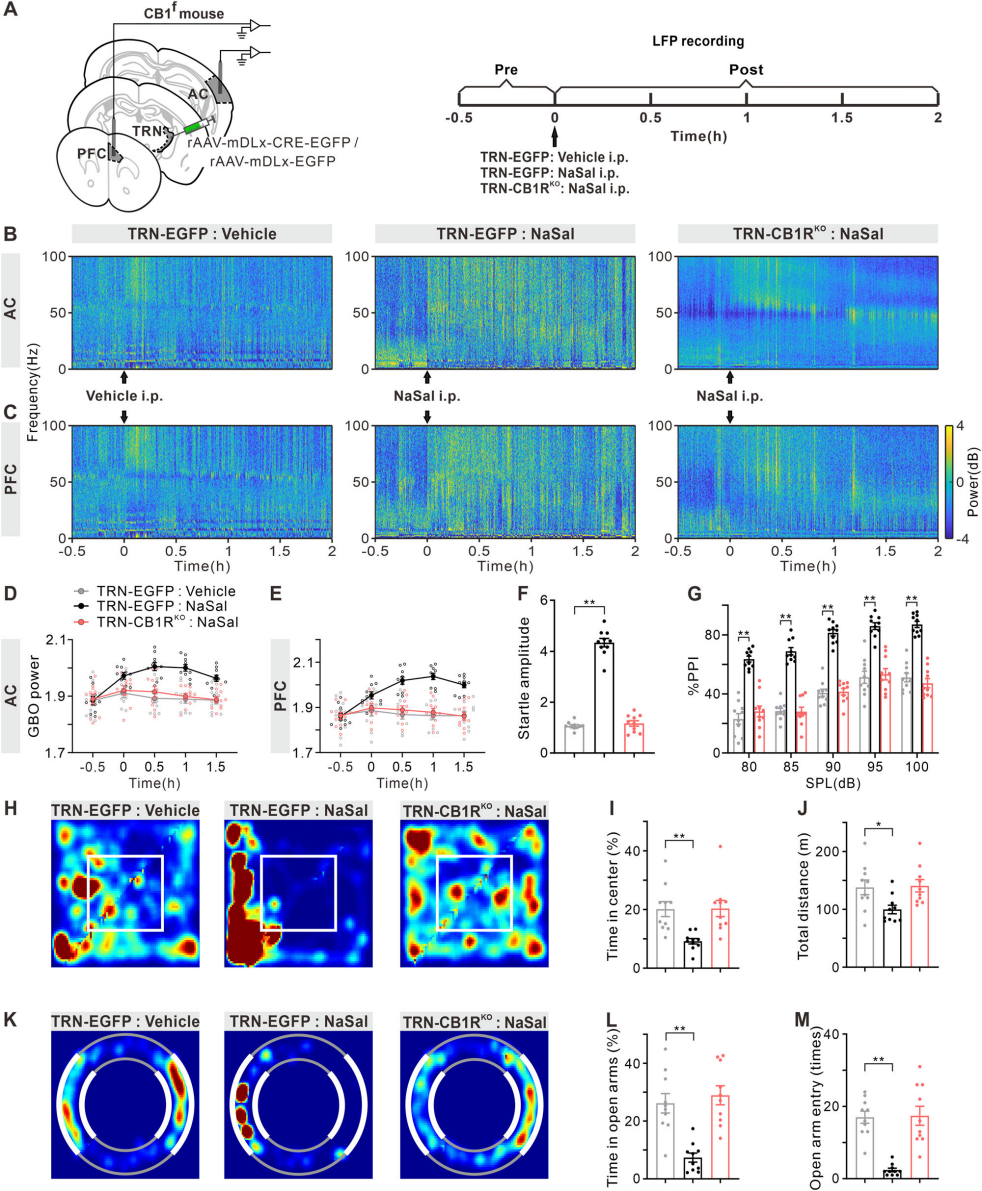
Absence of enhanced GBO and anxiety-like behaviors in NaSal-treated TRN-CB1R^KO^ mice. **A** Timeline of the virus injection, drug treatment and electrophysiological recording. **B**, **C** Example temporal-spectrograms of LFPs in the AC and PFC of TRN-EGFP or TRN-CB1R^KO^ mice received vehicle or NaSal injection. **D**, **E** Function of mean GBO power in the AC and PFC against time. **F** Startle amplitude to acoustic stimuli at 120 dB. **G** %PPI with different volumes of prepulse from 80 to 100 dB. **H** Representative occupancy heatmap showing spatial location in the OFT. **I** Time spent in the center of the arena. **J** Total traveled distance in the OFT. **K** Representative occupancy heatmap showing spatial location in the O-maze. **L** Time spent in open arms in the O-maze. **M** Number of entries into open arms in the O-maze. Error bars: SEM. *p < 0.05, **p < 0.01, one-way/two-way ANOVA with multiple comparisons test, *n* = 10 mice per group.

Considering that NaSal or AM251 may also impact local inhibitory circuits in brain regions such as the AC, PFC, or amygdala, it is necessary to further investigate the extent to which TRN CB1R plays a role in NaSal-induced GBO enhancement and behavioral abnormalities. For this, we injected rAAV-mDLx-CRE-EGFP into the TRN of CB1^f^ mice to selectively knock out CB1Rs (TRN-CB1R^KO^), and rAAV-mDLx-EGFP was used as a control (TRN-EGFP, Fig. 9A). NaSal-treated TRN-EGFP mice showed significantly prolonged GBO enhancement in the AC and PFC compared to vehicle-treated TRN-EGFP mice. In contrast, NaSal-treated TRN-CB1R^KO^ mice did not exhibit a pronounced enhancement in GBO (Fig. 9B-E, AC: F _treatment (2, 27)_ = 11.26, *p* = 0.0003, F _time (4, 108)_ = 14.60, *p* < 0.0001, F _treatment × time interaction (8, 108)_ = 7.11, *p* < 0.0001; PFC: F _treatment (2, 27)_ = 11.66, *p* = 0.0002, F _time (4, 108)_ = 51.50, *p* < 0.0001, F _treatment × time interaction (8, 108)_ = 43.49, *p* < 0.0001). Compared to vehicle-treated TRN-EGFP mice, NaSal-treated TRN-EGFP mice exhibited obvious increases in startle amplitude and %PPI in the ASR/PPI test (Fig. 9F and G), time spent in the center and total distance traveled in the OFT (Fig. 9H-J), time spent in the open arms and number of entries into the open arms in the O-maze test (Fig. 9K-M). On the contrary, there was no significant difference between NaSal-treated TRN-CB1R^KO^ mice and vehicle-treated TRN-EGFP mice (Fig. 9F, F _(2, 27)_ = 251.50, *p* < 0.0001; Fig. 9G, F _treatment (2, 135)_ = 293.40, *p* < 0.0001, F _prepulse intensity (4, 135)_ = 48.09, *p* < 0.0001, F _treatment × prepulse intensity interaction (8, 135)_ = 0.64, *p* = 0.7409; Fig. 9I, F _(2, 27)_ = 8.19, *p* = 0.0017; Fig. 9J, F _(2, 27)_ = 4.74, *p* = 0.0172; Fig. 9L, F _(2, 27)_ = 16.85, *p* < 0.0001; Fig. 9M, F _(2, 27)_ = 22.29, *p* < 0.0001). These results suggest that CB1R within the TRN plays a critical role in the cortical GBO enhancement and behavioral alterations induced by NaSal.

## Discussion

In this study, we investigated the neural and molecular mechanisms underlying hyperacusis and anxiety-like behaviors in NaSal-treated mice using behavioral tests, electrophysiological recording, fiber-optic recording, optogenetics, conditional gene knockout, and histological methods. We found the mice who underwent NaSal treatment exhibited hyperacusis and anxiety-like behaviors. This was accompanied by an increase in Fos expression and enhancement of GBO in both the AC and PFC. Additionally, NaSal increased the activity of glutamatergic neurons in the MGB and MD, as well as their phase-locking with the gamma bursts in the AC and PFC. By manipulating the MGB-AC and MD-PFC circuits, as well as TRN neurons, we could regulate the hyperacusis and anxiety-like behaviors. Further analysis revealed an elevation in the levels of eCBs in the MGB and MD of NaSal-treated mice, which acted on CB1Rs of TRN GABAergic neurons. The administration of AM251 reduced NaSal-induced GBO and behavioral abnormalities. The selective knockout of CB1Rs of TRN neurons also prevented NaSal-induced enhanced GBO and anxiety-like behaviors. These results emphasize the significance of eCBs/CB1Rs in GABAergic presynaptic inhibition and highlight the involvement of thalamic-cortical neural hyperactivation in hyperacusis and anxiety-like behaviors.

Previous studies have shown that NaSal has the ability to induce auditory perceptual disturbances (hyperacusis, tinnitus) in animals^70,71^. We validated the presence of these auditory abnormalities in mice by the ASR/PPI test. The NaSal-induced auditory abnormalities have been attributed to an increase in sound-evoked neural activity in the auditory processing centers, especially in the MGB and AC^70,72^. In addition, there is a suggested correlation between GBO in the auditory cortex and the presence of tinnitus^73^. GBO is an oscillatory pattern of neural activity commonly found in several brain regions^74–79^, and is thought to play an important role in sensory processing, attention, and memory^80–82^. In this study, we found that NaSal treatment enhances GBO in the AC. By the simultaneous electrophysiological recording of LFP and fiber-optic recording of neuronal calcium activity, we further found that NaSal injection enhanced the phase-locking between GBO bursts in the AC and calcium transients in the MGB neurons. Besides that, our optogenetic experiments confirmed that the activation of MGB-AC can induce hyperacusis in the ASR/PPI test, with no significant changes found in the O-maze test. In summary, NaSal-induced GBO increase in the AC is likely associated with the hyperactivation of MGB neurons, and the hyperexcitation of MGB-AC may further trigger hyperacusis in the mice.

Apart from causing transient hyperacusis and tinnitus, previous research has also documented that NaSal induces anxiety-like behaviors in animals by OFT and elevated plus maze tests^59,60^. Our current study also confirmed this with the usage of OFT and O-maze tests. The reason for NaSal-induced anxiety-like behaviors is thought to be associated with the increase of GBO in the limbic system, such as the PFC^60^. We verified that NaSal treatment amplified GBO in the PFC and enhanced phase-locking between GBO bursts in the PFC and calcium transients in the MD neurons. Fiber photometry in the O-maze apparatus further revealed an association between MD neuron activity and anxiety. In contrast to MGB-AC activation, activating MD-PFC can induce anxiety-like behaviors in the O-maze test, but no significant changes observed in the ASR/PPI test. Thus, NaSal-induced GBO increase in the PFC may be caused by the hyperactivity of MD neurons, and this could ultimately induce anxiety-like behaviors in the mice.

The prevailing view suggests that NaSal causes tinnitus or anxiety by disrupting the balance between excitatory and inhibitory neuronal activity in the brain. The inhibitory effect mediated by GABA is crucial for maintaining a balance of excitation and inhibition in the CNS^83,84^. Clinical experiments have indicated that the level of GABA in the AC is significantly reduced in patients with tinnitus^15^. In patients with anxiety disorders, GABA concentration is also reduced in the limbic system, including the PFC and anterior cingulate cortex^85^. Patch clamp experiments conducted on NaSal mice found decreased inhibitory currents in AC neurons^57^, indicating that the hyperexcitation may be caused by a reduction in GABAergic inhibitory function. Since the TRN is entirely composed of GABAergic neurons which provide inhibitory projections to several thalamic nuclei, including MGB and MD, alterations in neural activity within TRN may be a significant contributing factor to NaSal-induced inhibition of the GABA system. Our fiber photometry experiments revealed that NaSal injection led to an inhibition of GABAergic neuronal activity in the TRN, which may contribute to the hyperexcitation of MGB and MD. Furthermore, optogenetic activation of TRN GABAergic neurons suggests that increased inhibitory input from the TRN can result in the downregulation of cortical GBO and amelioration of the associated auditory and emotional abnormalities induced by NaSal. Therefore, the TRN may play a key role in modulating the functions of auditory and emotional neural circuits.

Because NaSal is an inhibitor of COX-2, which can degrade eCBs^66^, NaSal treatment might cause an increase in eCB levels. Our results from fiber-optic recordings of eCB2.0 fluorescence dynamics confirmed this in the MGB and MD. eCBs are signaling lipids that activate cannabinoid receptors^86–89^. Cannabinoid receptors are divided into two classes: CB1R, which is located primarily in the brain^90,91^, and cannabinoid 2 receptors (CB2R), which is located mainly in immune cells in the periphery^92^. In the CNS, CB1R is most abundant on certain GABAergic interneurons in regions such as the cortex and hippocampus^93–95^. It has been well established that CB1R expression highly concentrates on presynaptic terminals, where it mediates retrograde signaling of eCBs and functionally suppresses presynaptic GABA release^69,96,97^.

To investigate CB1R expression in TRN GABAergic neurons, we further performed FISH to label cnr1 mRNA and immunofluorescence staining to label GAD67 protein. We confirmed CB1R expression in TRN GABAergic neurons, which is consistent with results from in situ hybridization experiments conducted by the Allen Institute for Brain Science. Additionally, application of AM251, a CB1R antagonist, attenuated NaSal-induced GBO, hyperacusis, and anxiety-like behaviors. Although NaSal or AM251 may also impact local inhibitory circuits in the AC, PFC, or other anxiety-related regions, we selectively knocked out CB1Rs in TRN GABAergic neurons and found that NaSal injection no longer induced significant GBO enhancement and anxiety-like behaviors. This suggests that CB1R within the TRN is a key target involved in NaSal-induced aberrant behaviors.

Taken together, we propose a possible molecular mechanism underlying NaSal-induced hyperacusis and anxiety-like behaviors: NaSal increases the levels of eCBs by inhibiting the activity of COX-2, which mainly acts on the CB1Rs of GABAergic neurons in the TRN. The activation of eCBs/CB1Rs pathway suppresses presynaptic GABA release, leading to the disinhibition of neurons in the MGB and MD, the enhancement of GBO in the AC and PFC, and hyperacusis and anxiety-like behaviors in mice.

A study conducted intracranial recordings of the AC (i.e., electrocorticography) in a patient with severe tinnitus for 14 years, revealing an increase in gamma and theta activity in one of the eight implanted electrodes^98^. Although there are no invasive recordings in the human MGB currently, it is reasonable to believe that the MGB also plays a critical role in tinnitus pathology given its crucial role in integrating and processing multimodal upstream and downstream signals^99^. On the other hand, several clinical studies have reported exaggerated PFC and MD responses in patients with anxiety disorders^100–102^. The comorbidity of anxiety and tinnitus is common in clinical practice, but it remains uncertain whether anxiety causes tinnitus or if tinnitus causes anxiety. The results of our study suggest that anxiety and tinnitus may occur simultaneously due to the involvement of the TRN, which regulates both the auditory thalamo-cortical pathway associated with tinnitus and the limbic thalamo-cortical pathway associated with anxiety. Therefore, a definitive causal relationship between tinnitus and anxiety may not exist; instead, their comorbidity appears to stem from TRN dysfunction. Due to its deep location and small size, limited clinical data is available regarding research on the TRN. However, some researchers speculate that the TRN plays a crucial role in regulating functions such as emotions, higher order cognitive and sleep^41,103,104^. Acute cannabinoid overdose can cause feelings of panic and anxiety, hallucinations, and psychosis^105^. This indicates that the activation of CB1R can effectively decrease the release of inhibitory neurotransmitter GABA from neurons, leading to the hyperactivation of excitatory neurons. Consequently, the modulation of CB1R on the inhibitory function of TRN neurons may play a significant role in neuropsychiatric disorders such as tinnitus and anxiety.

We found the involvement of MGB-AC and MD-PFC neural hyperactivation in NaSal-induced auditory and emotional abnormalities, suggesting that thalamo-cortical hyperexcitation may underlie the potential neural mechanism for the comorbidity of tinnitus and anxiety disorders. Moreover, our study underscored the pivotal regulatory role of the TRN in the thalamo-cortical pathway and provided insights into a possible molecular mechanism involving eCBs/CB1Rs-mediated GABAergic presynaptic transmission. These findings point toward a novel direction for targeted therapy in managing comorbid tinnitus and anxiety disorders by enhancing the regulatory function of TRN.

Several limitations exist in our study. First, we did not directly assess tinnitus in the NaSal model mice because there is currently a lack of reliable behavioral or electrophysiological experimental methods to detect tinnitus in mice. Although the gap-prepulse inhibition (gap-PPI) experiment is used as a potential tool for assessing tinnitus in animals and humans, its results exhibit significant variability and cannot yet serve as a definitive criterion for tinnitus detection. Several studies have also reported abnormalities in gap-PPI in NaSal-treated mice^106,107^. It is worth noting that previous clinical research has frequently observed concurrent occurrences of tinnitus and hyperacusis^108,109^. Given that the detection of hyperacusis is relatively straightforward and reliable, in our study we used the ASR/PPI experiment to assess hyperacusis in NaSal-treated mice, which to some extent may indicate the possibility of tinnitus in this model. However, whether and to what extent NaSal induces tinnitus in mice requires future application of more reliable detection methods for confirmation. Secondly, our current findings show a correlation between hyperacusis and anxiety-like behaviors in NaSal-treated mice, but do not establish a direct causal relationship between them. Moreover, anxiety and tinnitus may co-occur due to some shared neuropathological mechanisms. This issue warrants further investigation for clarification in future research. Finally, we employed a 1 s ON-1s OFF stimulation pattern during the TRN activation experiment to reduce the risk of photodamage. However, this intermittent stimulation protocol may have only partially restored TRN neuronal activity, failing to reach its physiological baseline. Therefore, mice still exhibited some degree of anxiety, which does not fully prove the role of TRN in regulating auditory hypersensitivity and anxiety.

## Methods

### Animals and drugs treatment

All animal experimental protocols were approved by the Animal Ethics Committee of China Medical University in accordance with Institutional Animal Care and Use Committee guidelines for animal research. We have complied with all relevant ethical regulations for animal use. C57BL/6 mice (Vital River Laboratory, Beijing, China), Fos^CreER^ mice (B6.129(Cg)-Fos^tm1.1(cre/ERT2)Luo^/J, #021882), Ai14 mice (B6.Cg-Gt(ROSA) 26Sor^tm14(CAG-tdTomato)Hze^/J, #007914), GAD2-cre mice (Gad2^tm2(cre)Zjh^/J, #010802) and CB1^f^ mice (B6.129P2(Cg)-Cnr1^tm1.2Ltz^/J, #036107) (Jackson Laboratory, ME, USA) were used. The FosTRAP mice were obtained from crossing female Ai14 mice with male Fos^CreER^ mice. All nomenclature and abbreviations for genotype combinations of transgenetic mice used in this study were listed in Supplementary Table 1.

All experiments were conducted using male mice aged 6 to 8 weeks, with a weight range of 20 to 25 g. Mice were housed in standard animal cages with sufficient food and water individually under controlled conditions (12 h light/12 h dark schedule, 22 ± 1°C, 30–50% humidity). All of the surgeries were performed under anesthesia to minimize the pain of animals.

For NaSal treatment, mice received an intraperitoneal (i.p.) injection of 300 mg/kg NaSal (HY-B0167A, MedChemExpress, NJ, USA) freshly diluted in 0.9% saline or an equal volume of saline (vehicle). For CB1 receptor antagonist AM251 treatment, AM251 (HY-15443, MCE) was first dissolved in 10% DMSO and then in 0.9% saline for i.p. administration at a final dose of 20 mg/kg.

### Behavioral test

#### ASR/PPI test

ASR/PPI tests were conducted on the free-moving mice in standard startle chambers. One block for PPI testing consists of 6 different sound stimuli with 10 repetitions: 1) pulse-alone: 40 ms white noise burst (120 dB); 2–6) prepulse-pulse: 20 ms white noise burst (80, 85, 90, 95, or 100 dB) + 100 ms inter-stimuli interval (ISI) + 40 ms white noise burst (120 dB). Trails were presented in randomized order, with 25–30 s randomized inter-trial interval.

#### OFT

The OFT was performed in an open field apparatus (54 × 40 × 30 cm) constructed of non-transparent white boards for well position tracking. Before testing, each mouse underwent a 5-min habituation in the procedure room. Then the mouse was placed into the center of the arena, and was allowed to explore for 30 min. Activity of the mouse was monitored via an overhead camera (C270 HD Webcam, Logitech). The open field apparatus was cleaned with 70% ethanol between each mouse.

#### Home cage spontaneous activity

Mice were observed in their home cages by video recording for 30 min. The testing took place 3 h after the beginning of the dark cycle.

#### O-maze

The elevated O-maze was a modification of the elevated plus maze. The elevated circular platform (100 cm off the ground, 50 cm in diameter) had two enclosed arms opposite each other (10 cm wide with 12 cm high walls) and two open arms (10 cm wide). Briefly, each mouse was gently lowered by its tail into the open arena of the maze and given 9 min to explore. For the O-maze involving optogenetic manipulation of TRN, the exploration period lasted 12 min. Because the simulations in the TRN involved continuous optogenetic activation, the exploration period was split into six 2-min sections (OFF-ON-OFF-ON-OFF-ON).

For O-maze with MGB-AC and MD-PFC optogenetic manipulation, the exploration duration was 9 min, and for O-maze with TRN optogenetic manipulation, the exploration duration was 12 min. The whole circular platform was cleaned with 70% ethanol and wiped with paper towels between each animal.

#### Fos reporter activation

4-OHT was dissolved in ethanol to 20 mg/mL by shaking at 37 °C for 15 min, then aliquoted and stored at −20 °C for up to a month. Before use, 4-OHT was re-dissolved in ethanol by shaking at 37 °C for 15 min. Corn oil (HY-Y1888, MCE) was added to give a final concentration of 5 mg/mL, and the ethanol was evaporated by vacuum under centrifugation.

FosTRAP mice were given 7 days for the habituation of daily handling and i.p. injection. On day 8, the mice received an i.p. injection of 4-Hydroxytamoxifen (4-OHT, HY-16950, MCE 50 mg/kg)^110,111^, and another injection of vehicle/NaSal 30 min later. Mice were kept in their home cage for 72 h and then euthanized on day 11 for perfusion and immunohistochemistry analysis.

#### Tissue processing and immunohistochemistry

Mice were anesthetized and transcardially perfused with PBS and 4% PFA. Brains were dissected, postfixed in 4% PFA, and dehydrated in gradient sucrose. Embedded in Tissue-Tek O.C.T compound (Sakura Finetek, CA, USA), frozen brains were cut on a cryostat (Leica CM 1900, Leica Biosystems, Nussloch, Germany) into 15 μm-thick sections.

For immunostaining, sections were incubated in normal goat serum and primary antibodies (Anti-NeuN, 1:500, ab177487, Abcam, MA, USA; GAD67/Gad1 antibody, 1:300, A2938, ABclonal, Wuhan, China) overnight at 4 °C, followed by the appropriate fluorescent secondary antibody (1:300, Proteintech, Wuhan, China).

Combined FISH for *Cnr1* and GAD67/Gad1 with immunostaining was conducted. The FAM-labeled *Cnr1* probe (GenePharma, Shanghai, China) was used to detect *Cnr1* in GAD67/Gad1-labeled cells. Briefly, FISH was performed in the frozen sections by using FISH kit (GenePharma) according to the manufacturer’s protocol. The slides were then washed and blocked for the immunostaining, and mounted in an anti-fade reagent with DAPI (SL1841, Coolaber, Beijing, China). All abbreviations of gene names and protein/receptor names in this study were listed in Supplementary Table 2.

Tissue sections were observed using a fluorescence microscope (BX53, Olympus, Tokyo, Japan). Cell counts for Fos and NeuN were obtained with IMARIS software (Bitplane, Zürich, Switzerland). The borders of regions were defined manually according to the Franklin and Paxinos mouse brain atlas^112^. All imaging and analyses were conducted in a blinded manner, without knowledge of the experimental conditions.

#### Surgeries and viral injections

##### Stereotaxic surgical procedures

Mice were anesthetized with isoflurane (3–4% isoflurane for induction, 1–2% for maintenance) and placed in a stereotaxic head frame (68001, RWD Life Science, Shenzhen, China). Sterile eye ointment was applied to prevent corneal drying, and a heating pad was used to maintain body temperature. The fur on the head was removed and the skin was triple scrubbed with 70% ethanol. The skin was excised from the head, and then the skull was gently scraped clean with a bone scraper.

##### Viral injections

Glass pipettes were pulled and beveled to a sharp tip, and a syringe pump (Pump 11 Elite, Harvard Apparatus, MA, USA) was used to control the infusion. We injected 300 nl of virus into each location (MGB: AP = −3.2 mm, ML = + 2.0 mm, DV =  −3.2 mm; MD: AP =  −1.6 mm, ML = + 0.5 mm, DV =  −3.5 mm; TRN: AP =  −1.1 mm, ML = + 2.0 mm, DV =  −3.2 mm) at a rate of 100 nl/min. The needle was kept in situ for an additional 10 min after the virus was fully injected and then slowly withdrawn.

For fiber-optic recordings, rAAV-CaMKIIa-GCaMP6s-WPRE-hGH pA (PT-0110, BrainVTA, Wuhan, China) or rAAV-hSyn-GRAB_eCB2.0-WPRE-hGH polyA (PT-3571) was injected into unilateral MGB and MD. rAAV-CAG-DIO-GCaMP6s-WPRE-hGH polyA (PT-0196) was injected into unilateral TRN of GAD2-cre mice.

For in vivo optogenetic activation experiments, mice were unilateral injected with rAAV-CaMKIIa-hChR2(E123T/T159C)-EYFP-WPRE-hGH polyA (PT-0004) to activate the MGB or MD glutamatergic neurons. rAAV-CAG-DIO-EYFP-WPRE-hGH polyA (PT-0806, EYFP group) or rAAV-CAG-DIO-hChR2(H134R)-EYFP-WPREs (PT-0474, ChR2 group) were injected into GAD2-cre mice to activate TRN GABAergic neurons.

To specifically knock out CB1Rs in TRN neurons, mice received bilateral injections of rAAV-mDlX-CRE-EGFP-WPRE-hGH polyA (PT-2267) into the TRN.

##### Electrode, fiber and optrode implantation

The electrodes, fibers or optrodes were implanted in the AC (AP = −2.2 mm, ML = + 3.7 mm, DV = −1.8 mm), PFC (AP = + 1.7 mm, ML = + 0.5 mm, DV = −2.2 mm), MGB, MD or TRN.

For simultaneous recordings of LFP and calcium activity, a custom-built optrode, consisting of an optical fiber (200 µm diameter, 0.37 NA, Newdoon, Hangzhou, China) surrounded by 4 formvar-insulated nichrome microwire electrodes (762000, A-M Systems, Hofheim, USA) twisted into stereotrodes, was implanted 200 µm above the viral injection site. The other end of the microwire was soldered to a pin connector, which was secured onto the cranium of the right hemisphere using dental cement. The same optrode was also used in the optogenetic experiments and LFP recording. Two stainless-steel screw electrodes were also implanted above the cerebellum as a ground and reference.

For electrophysiological recordings, a stainless-steel guide tube (30 G) was inserted into the PFC and AC of left hemisphere. After fixing the tube with dental cement, we lowered microwire electrodes into the tube and kept the tip at the level of 500 µm below the opening of the tube. Additionally, four skull screws were implanted as anchors, while two screw electrodes were used as ground and reference.

For fiber-optic recordings of eCB2.0, an optical fiber was implanted 200 µm above the viral injection site.

Afterwards, dental cement was used to adhere them to the skull. Animals were allowed to recover for at least 2 weeks.

##### **I**n vivo electrophysiological recording

The mice were accustomed to head fixation and familiarized with the environment for 1 h where they were allowed to move in place freely on a spinning disk. For electrophysiological recordings, a low-noise flexible cable was connected to the connector implanted on the skull. The raw signals were digitized with a multichannel extracellular amplifier (RA16PA, Tucker-Davis Technologies, FL, USA). And they were band-pass filtered to extract LFP (1–300 Hz), then imported into computer for further analysis. We firstly recorded the LFPs for 0.5 h before vehicle or NaSal injection (Pre), subsequently recorded a 3 h or 1 h period after the injection (Post).

##### **F**iber-optic recording

Fiber-optic recording was performed 3 weeks after viral injections to allow for expression of the transgene. A fiber optic cable was firmly attached to the implanted fiber optic cannula. The optical-fiber recording was carried out by a fiber photometry system (R811, RWD). In brief, the 470 and 410 nm laser beams first launched into the fluorescence cube, then launched into the optical fibers. 410 laser was used for motion control. The GCaMP6s, eCB2.0 and control emission fluorescence were collected by the camera at 15 Hz. Fiber-optic recording of 3 min was performed three times with an interval of 10 min at 0.5 h pre-injection and 1 h post-injection. For recordings in the O-maze apparatus, mice with a fiber optic cable connected to the fiber photometry system were allowed to freely explore in the O-maze for a period of 3 min.

##### **I**n vivo optogenetic manipulation

Animals with optical fiber implants were connected to an optical patch cable via a ceramic sleeve (Newdoon) coupled to a 470 nm laser (Aurora-300, Newdoon). Laser at 470 nm was applied to animals that expressed ChR2 and their respective EYFP controls.

##### **A**nalysis of behavioral data

PPI data were presented as percentage of PPI (%PPI), which was calculated from ASR using the following formula: %PPI = 100 − [(ASR for prepulse-pulse trials)/(average ASR for pulse-alone trials)×100].

Mouse movement was identified by a custom-written software in MATLAB (R2018a, The Mathworks, MA, USA). The center of the arena was defined as the central 27 × 20 cm portion. Time spent in the center arena and total traveled distance were determined.

For the O-maze tests, mouse movement was also identified by the custom MATLAB script. Time spent in the open arms and number of entries into the open arms were measured. For optogenetic activation studies, a third indicator, the self-grooming time, was measured. Grooming behavior encompassed activities such as head washing, body grooming, genital/tail grooming, as well as paw and leg licking. A trained observer recorded the total duration of self-grooming.

##### **A**nalysis of LFP and fiber photometry data

The power spectrogram of LFP signals was calculated using the “mtspecgramc” function in the Chronux toolbox (http://chronux.org/)^113^. To compensate for power attenuation with frequency, power was calculated as its logarithm. The mean power at gamma band was calculated by averaging the power across the frequency bins within 30–80 Hz.

The datasets of fiber-optical recording from each mouse were pooled for analysis. We calculated the optical signal value F as F470/F410 and then determined ∆F/F = (F – F0) / F0, with F0 representing the median of the optical signal. A transient event was defined as a change in the ΔF/F value that exceeded at least one standard deviation (SD) above the mean value of the ΔF/F during the recording period before injection. The amplitude of transient event was determined by calculating the difference between the peak and mean values. Subsequently, the mean of transient amplitude and frequency was computed for each mouse.

For the analysis of fiber-optical recording in the O-maze apparatus, the Z-score of the optic signal was calculated using the formula: Z = (x-y)/SD (where x = ∆F/F_Signal_, y = mean of ∆F/F_Baseline_, and SD for standard deviation of ∆F/F_Baseline_).

For the analysis of LFP data under optogenetic activation, we aligned the recorded LFP to the onset of the optogenetic stimulus for every trial, and extracted the recording epochs using a window ranging from 200 ms before to 1800 ms after stimulus onset. The LFP signals were corrected by subtracting 200-ms pre-stimulus baseline from the post-stimulus wave. Then the power spectrogram and mean GBO power of corrected LFP signals were calculated.

##### **S**tatistics and reproducibility

Statistical analysis was performed using GraphPad Prism 8.0 (GraphPad, CA, USA). The normally distributed data were analyzed by unpaired *t*-test or one-way ANOVA with Tukey multiple comparisons test. %PPI and mean GBO power were analyzed by two-way ANOVA with Sidak multiple comparisons test. The locomotor activity parameters in the O-maze under optogenetic activation were analyzed by one-way ANOVA with Geisser-Greenhouse correction and Dunnett multiple comparisons test. Results were considered statistically significant when the *p* value < 0.05.

For reproducibility, all experiments were performed with a minimum of 5 biological replicates, defined as independent experiments conducted under the same conditions. Sample sizes for each experiment were determined based on prior studies and power analysis to ensure adequate statistical power. All data are reported as mean ± SEM, and detailed information on sample sizes and replicates is provided in the respective sections of the Results.

### Reporting summary

Further information on research design is available in the Nature Portfolio Reporting Summary linked to this article.

## Supplementary information

Supplementary Information

Description of Additional Supplementary Files

Supplementary Data 1

Reporting Summary

## Data Availability

The data that support the findings of this study are available from Supplementary Data 1.

## Code availability

The custom codes used for analyzing data are available at https://github.com/JingyuChen96/FiberRecordingAndBehavioralAnalysis.
