# Supplementary material for: Thalamo-cortical neural mechanism of sodium salicylate-induced hyperacusis and anxiety-like behaviors
Source: Commun Biol. 2024 Oct 18;7:1346. doi: 10.1038/s42003-024-07040-5 (PMC11487285; doi:10.1038/s42003-024-07040-5)
Supplement: Supplementary file 1 — Supplementary Information [file 42003_2024_7040_MOESM1_ESM.pdf]

**Supplementary Table 1**<br>Nomenclature and abbreviations for genotype combinations of transgenetic mice	
**Abbreviation**	**Genotype**
Fos^{CreER}	B6.129(Cg)-$Fos^{tm1.1(cre/ERT2)Luo}$/J
Ai14	B6.Cg-Gt(ROSA) $26Sor^{tm14(CAG-tdTomato)Hze}$/J
FosTRAP	B6.129(Cg)-$Fos^{tm1.1(cre/ERT2)Luo}$/J × B6.Cg-Gt(ROSA) $26Sor^{tm14(CAG-tdTomato)Hze}$/J
GAD2-cre	$Gad2^{tm2(cre)Zjh}$/J
$CB1^{f}$	B6.129P2(Cg)-$Cnr1^{tm1.2Ltz}$/J

**Supplementary Table 2**<br>Abbreviations of gene names and protein/receptor names	
**Abbreviation**	**Full name**
Cnr1	Gene symbol of cannabinoid receptor 1
CB1R	Protein/receptor name of cannabinoid receptor 1
Fos	FBJ osteosarcoma oncogene
Gad1	Gene symbol of glutamate decarboxylase 1
GAD67	Protein name of glutamate decarboxylase 1
