# Supplementary material for: Thalamo-cortical neural mechanism of sodium salicylate-induced hyperacusis and anxiety-like behaviors
Source: Commun Biol. 2024 Oct 18;7:1346. doi: 10.1038/s42003-024-07040-5 (PMC11487285; doi:10.1038/s42003-024-07040-5)
Supplement: Supplementary file 2 — Description of Additional Supplementary Files [file 42003_2024_7040_MOESM2_ESM.pdf]

Description of Additional Supplementary Files

File name: Supplementary Data 1

Description: The source data behind the graphs in the paper.
